# Dopamine D3 Receptor Mediates Preadolescent Stress-Induced Adult Psychiatric Disorders

**DOI:** 10.1371/journal.pone.0143908

**Published:** 2015-11-30

**Authors:** Joon H. Seo, Eldo V. Kuzhikandathil

**Affiliations:** Department of Pharmacology, Physiology and Neurosciences, Rutgers-New Jersey Medical School, Newark, NJ, 07103, United States of America; Roma Tre University, ITALY

## Abstract

Several studies have shown that repeated stressful experiences during childhood increases the likelihood of developing depression- and anxiety-related disorders in adulthood; however, the underlying mechanisms are not well understood. We subjected *drd3*-EGFP and *drd3*-null mice to daily, two hour restraint stress episodes over a five day period during preadolescence (postnatal day 35 to 39), followed by social isolation. When these mice reached adulthood (post-natal day > 90), we assessed locomotor behavior in a novel environment, and assessed depression-related behavior in the Porsolt Forced Swim test. We also measured the expression and function of dopamine D3 receptor in limbic brain areas such as hippocampus, nucleus accumbens and amygdala in control and stressed *drd3*-EGFP mice in adulthood. Adult *male* mice subjected to restraint stress during preadolescence exhibited *both* anxiety- and depression-related behaviors; however, adult *female* mice subjected to preadolescent restraint stress exhibited *only* depression-related behaviors. The development of preadolescent stress-derived psychiatric disorders was blocked by D3 receptor selective antagonist, SB 277011-A, and absent in D3 receptor null mice. Adult male mice that experienced stress during preadolescence exhibited a loss of D3 receptor expression and function in the amygdala but not in hippocampus or nucleus accumbens. In contrast, adult female mice that experienced preadolescent stress exhibited increased D3 receptor expression in the nucleus accumbens but not in amygdala or hippocampus. Our results suggest that the dopamine D3 receptor is centrally involved in the etiology of adult anxiety- and depression-related behaviors that arise from repeated stressful experiences during childhood.

## Introduction

The transition from adolescence to adulthood is associated with many behavioral changes. In humans and rodents repeated or chronic stress during preadolescence has long-lasting effects and result in development of behavioral disorders as adults [1–9]. In a Center for Disease Control study published in 2008, a survey of 17,000 adults revealed that ~64% had experienced one or more adverse childhood experience [3]. The number of adverse childhood experience correlated to attempted suicides, substance abuse and depressive disorders in adulthood [1–9]. Molecular mechanisms by which adverse childhood experiences cause psychiatric disorders in adults are not well understood.

Studies have shown that stress induces dopamine release in the limbic regions [10–12]. The limbic regions express five different dopamine receptor subtypes, including the dopamine D3 receptor which has the highest affinity for dopamine. We and others have shown that the D3 receptor couples to multiple signal transduction cascades both *in vitro* and *in vivo*, including the mitogen-activated protein kinase (MAPK) pathway [13]. We have also reported that repeated activation of D3 receptor induces a tolerance property which results in progressive loss of D3 receptor signaling function [13]. The D3 receptor is a clinically relevant therapeutic target for depression and anxiety-related behaviors as clinically-used D3 receptor-preferring agonists, aripiprazole, pramipexole and ropinirole, have all been shown to have antidepressant and anxiolytic effects in humans and animals models [14–17]. Several D3 receptor ligands are being currently evaluated for treating depression [17]. Adult D3 receptor null mice have also been shown to be resistant to the behavioral dysfunction that results from immobilization stress [18–20]. Recently, D3 receptor was also shown to modulate anxiety-like behaviors and GABAergic neurotransmission in the lateral/basolateral amygdala [21]. However no studies have compared the role of dopaminergic system in the amygdala in preadolescent and adult animals and investigated the role of dopamine D3 receptors in the behavioral changes that occur during the transition from adolescence to adulthood in males and females. Here we tested the hypothesis that adult anxiety- and depression-related disorders induced by preadolescent repeated restraint stress and social isolation is mediated by the dopamine D3 receptor. The hypothesis was tested using *drd3*-EGFP reporter mice which express the enhanced green fluorescent protein (EGFP) in cells that express endogenous D3 receptor mRNA, as well the *drd3* null mice which does not express the D3 receptor protein. The genetic knockout studies were complemented with pharmacological studies using a dose of D3 receptor antagonist, SB277011-A, which we have recently shown to selectively block the D3 receptor-MAPK signaling pathway in the *drd3*-EGFP mice [13].

## Materials and Methods

### Mice

The *drd3*-EGFP mice and the *drd3*-null mice were kindly provided by Drs. Mary Beth Hatten and Nathaniel Heintz (GENSAT program, Rockefeller University), and Dr. Neil Richtand (University of California, San Diego), respectively. Local breeding colonies for both strains were established at Rutgers-New Jersey Medical School. The *drd3*-EGFP reporter mice are on a mixed Swiss Webster/FVB genetic background and have been recently characterized by us in detail [13, 22]. The *drd3*-null mice are on a C57BL/6 background and have been previously characterized as well [23, 24]. The mice were weaned on post-natal day 21 (P21) and group housed (4 or less mice per cage) with littermates until the beginning of experiments. The mice were housed in a temperature and humidity controlled environment, with a 12 hour light (on at 7 A.M.): dark (off at 7 P.M.) cycle. All experiments were performed in the light phase. Mice were provided *ad libitum* access to standard rodent chow and water. All procedures were approved by the IACUC committee at Rutgers-New Jersey Medical School.

### Preadolescent repetitive restraint stress

On post-natal day 35 (P35), the mice were divided into two groups. The non-stressed mice were group housed. The mice subjected to preadolescent restraint stress were individually placed in clear 50 ml polypropylene tube with air vents at the nose and tail. The diameter of the tubes did not allow the mice to turn or twist around. The tubes with mice were placed on a lab bench in a lighted room. The restraint stress was performed for five consecutive days (P35 to P39), 2 hours per day, at random times between 9 AM and 2 PM. During and after the restraint stress episodes, the mice were single-housed in a vivarium room with other mouse colonies. In experiments involving pharmacological antagonism of D3 receptor, the mice were administered saline or 10 mg/kg SB277011-A, intraperitoneally, ten minutes before being subjected to the restraint stress episode over the 5-day period. All mice were behaviorally and biochemically tested as adults (>P90).

### Open field test

Horizontal locomotor activity was measured using the open field photo beam activity system (PAS; SD Instruments, San Diego, CA, USA) as described by us recently [13]. The PAS open field arena has an inner dimension of 16 inch x 16 inch with photo beams that are 1 inch apart in each direction. The center zone was defined as a 8 inch X 8 inch area in the center of the arena and a center zone entry was defined as two photobeam breaks in the center zone. Photobeam breaks were converted to total distance traveled in cm using the PAS reporter software (version 2). The resting time parameter in the software was set at 4 seconds. Mice were brought into the procedure room and, within approximately five minutes, placed close to the interior side of the wall in the open field arena. Data collection commenced immediately and continued for 60 minutes.

### Porsolt forced swim test

The mice were individually placed in glass cylindrical containers (15 cm diameter, 50 cm height) filled with 25 cm deep water (temp 22°C–25°C) for six minutes. The entire six minute duration was recorded with a video recorder. Latency to first immobility and total immobility time were measured during the last four minutes of the six-minute recording [25]. The total duration of immobility was measured and defined as the summary of time spent in all immobility bouts in the last four minutes of the six-minute period [25]. A mouse was judged immobile when it ceased all active behaviors (i.e., struggling, swimming and jumping) and remained passively floating and making minimal movements necessary to maintain the nostrils above water. The minimal duration of a bout of immobility was set at five seconds without any active escape behavior. After each test, the mouse was taken out and quickly dried with paper towels and returned to home cage.

### Drug administration

D3-dopamine receptor agonist R-(+)-trans-3,4,4a,10b-tetrahydro-4-propyl-2 H,5 H-(1)benzopyrano[4,3-b]-1,4-oxazine-9-ol (PD128907; Tocris, Minneapolis, MN, USA) was used to assess the signaling function of the D3 receptors. The drug was dissolved in saline and injected subcutaneously at 0.05 mg/kg concentration ten minutes before brain tissue collection. D3 receptor antagonist, N-[trans-4-[2-(6-Cyano-3,4- dihydro-2(1H)-isoquinolinyl)ethyl]cyclohexyl]-4-quinolinec-arboxamide dihydrochloride (SB277011-A; Tocris), was dissolved in saline and then injected intraperitoneally at 10mg/kg, ten minutes before the induction of preadolescent restraint stress. We and others have shown that the dose of SB277011-A and PD128907 used in the experiments are selective for D3 receptors [13, 26].

### Tissue harvest and Western blotting

To determine the function of D3 dopamine receptor, both stressed mice and non-stressed *drd3*-EGFP mice were injected with either saline or PD128907 (0.05 mg/kg), 10 minutes before brain tissue collection. A brain block was used to obtain 0.5 mm thick coronal sections of the brain. Individual brain region was dissected out of the coronal sections under a fluorescence dissecting microscope with appropriate EGFP filters; this approach allowed isolation of tissue enriched in D3 receptor-expressing cells. All tissue were kept at -80°C until lysis which was carried out as described previously [13]. Levels of phosphorylated and total ERK proteins and D3 receptors were determined by western blotting as described previously [13].

### Data analysis

All statistical analysis was performed using SigmaPlot (version 11). Reported numbers of replicates (n values) represent numbers of mice used in each experiment. The total number of mice indicated in the figure legends were divided equally among the various treatment groups in each experiment shown in the figure legend. The data were plotted as the mean and error bars represent standard error of the mean. No formal statistical calculations were used to pre-determine sample sizes. When data distribution was normal with similar variance between the groups, parametric analysis for statistical comparison was used. Otherwise non parametric tests such as Mann–Whitney U test and Kruskal–Wallis test were used. All statistical tests were two-tailed. No blinding was performed during experiments or analysis. To isolate groups that were statistically different, ANOVA or repeated measure ANOVA was performed, followed by post-hoc multiple comparison tests, including Holm-Sidak and Student Neuman Keuls (SNK) tests, as described in the figure legends and text. P<0.05 was considered statistically significant.

## Results

### Repeated restraint stress during preadolescence and social isolation causes anxiety-related disorders in adult male but not female mice

**Fig 1:**
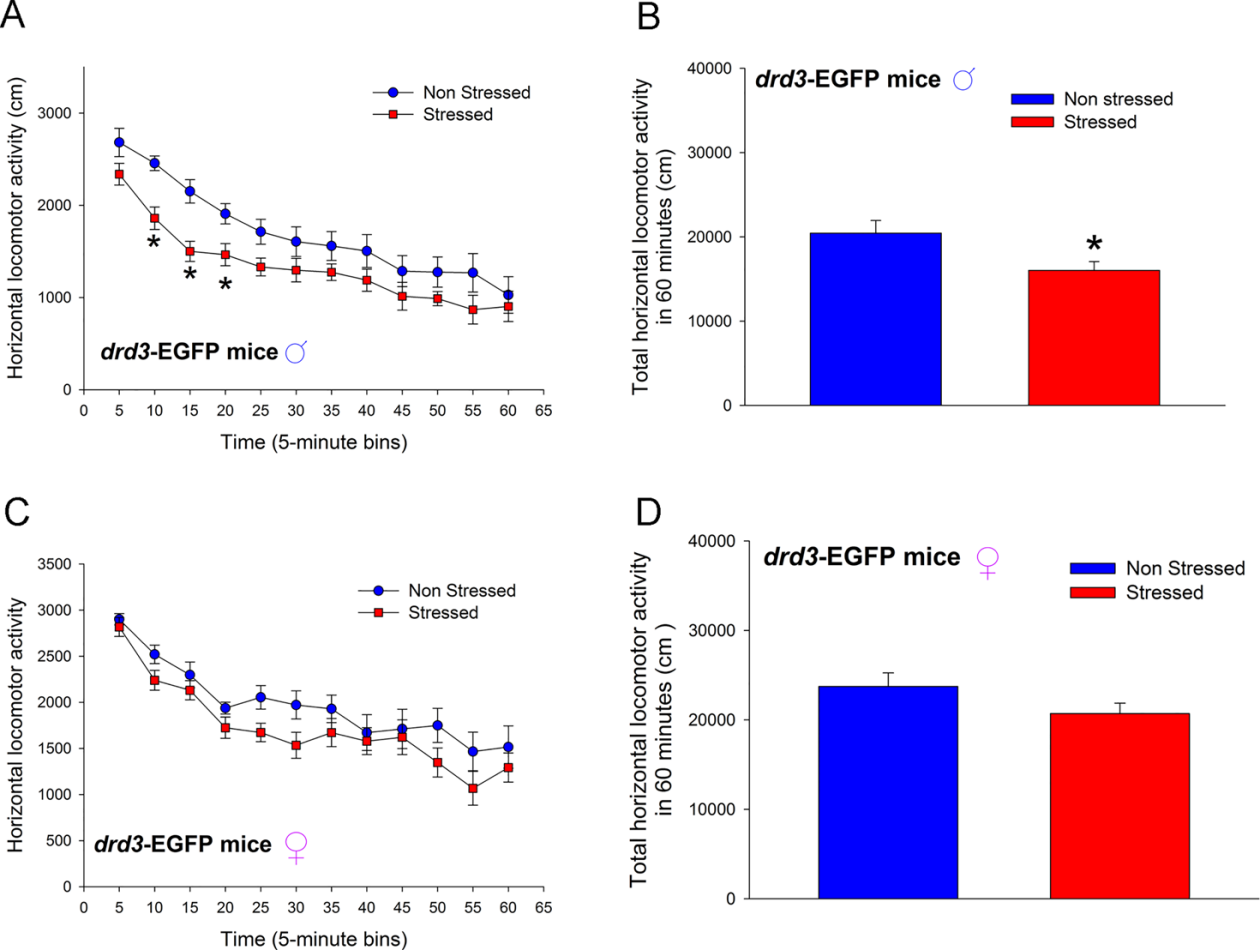
Effect of repeated restraint stress and social isolation during preadolescence on locomotor activity in an open field arena by adult *drd3*-EGFP mice. Adult male (**A** and **B**) and female (**C** and **D**) *drd3*-EGFP mice were either not stressed (blue, n = 11) or subjected to repeated restraint stress and social isolation during preadolescence (red, n = 11). Horizontal locomotor activity was measured in an Open Field test in adulthood. Significant reduction in locomotor activity was observed in stressed male *drd3*-EGFP mice during the 10, 15 and 20 minute intervals following the placement of the mice in the arena (*, p<0.05, two-way repeated measure ANOVA, post-hoc SNK test; **A**). The total locomotor activity over the entire 60 minute observation period was also significantly reduced in stressed male *drd3*-EGFP mice (*, p<0.05, Student’s *t*-test). The stress-induced changes in locomotor activity was absent in adult female *drd3*-EGFP mice subjected to preadolescent stress and social isolation (**C** and **D**). Error bars represents ± SEM.

Stressed mice were subjected to daily two hour restraint stress over five consecutive days from postnatal day 35 (P35) to P39, followed by social isolation whereas non-stressed mice were never subjected to restraint stress or social isolation. All behavioral and biochemical assessments were carried out in adulthood when the mice were >P90. Male *drd3*-EGFP mice that were subjected to preadolescent repetitive restraint stress followed by social isolation exhibited, in adulthood, a significant reduction in locomotor activity in a novel environment (*p* = 0.021, F_1,242_ = 6.166, two-way repeated measure ANOVA; Fig 1A) wherein, post-hoc SNK test revealed significant difference between the non-stressed and stressed mice only at the 10 min (*q* = 4.274, *p* = 0.004), 15 min (*q* = 4.667, *p* = 0.002) and 20 min (*q* = 3.185, *p* = 0.028) time points. No significant difference in basal locomotor activity was observed during the initial 5 min period after the mice were placed in the novel arena (*q* = 2.475, *p* = 0.085). The large decrease in locomotor activity during the 10 to 20 min interval resulted in an overall decrease in locomotor activity over the entire 60 minute monitoring period (*p* = 0.021, Student’s t-test; Fig 1B). Female *drd3*-EGFP mice that were subjected to preadolescent repetitive restraint stress followed by social isolation did not exhibit, in adulthood, a significant reduction in locomotor activity in a novel environment (*p* = 0.144, F_1,165_ = 2.380, two-way repeated measure ANOVA; Fig 1C) and, post-hoc SNK test did not reveal significant difference between the non-stressed and stressed mice at any time points. There was also no significant difference in locomotor activity during the entire 60 minute monitoring period (*p* = 0.144, Student’s t-test; Fig 1D).

**Fig 2:**
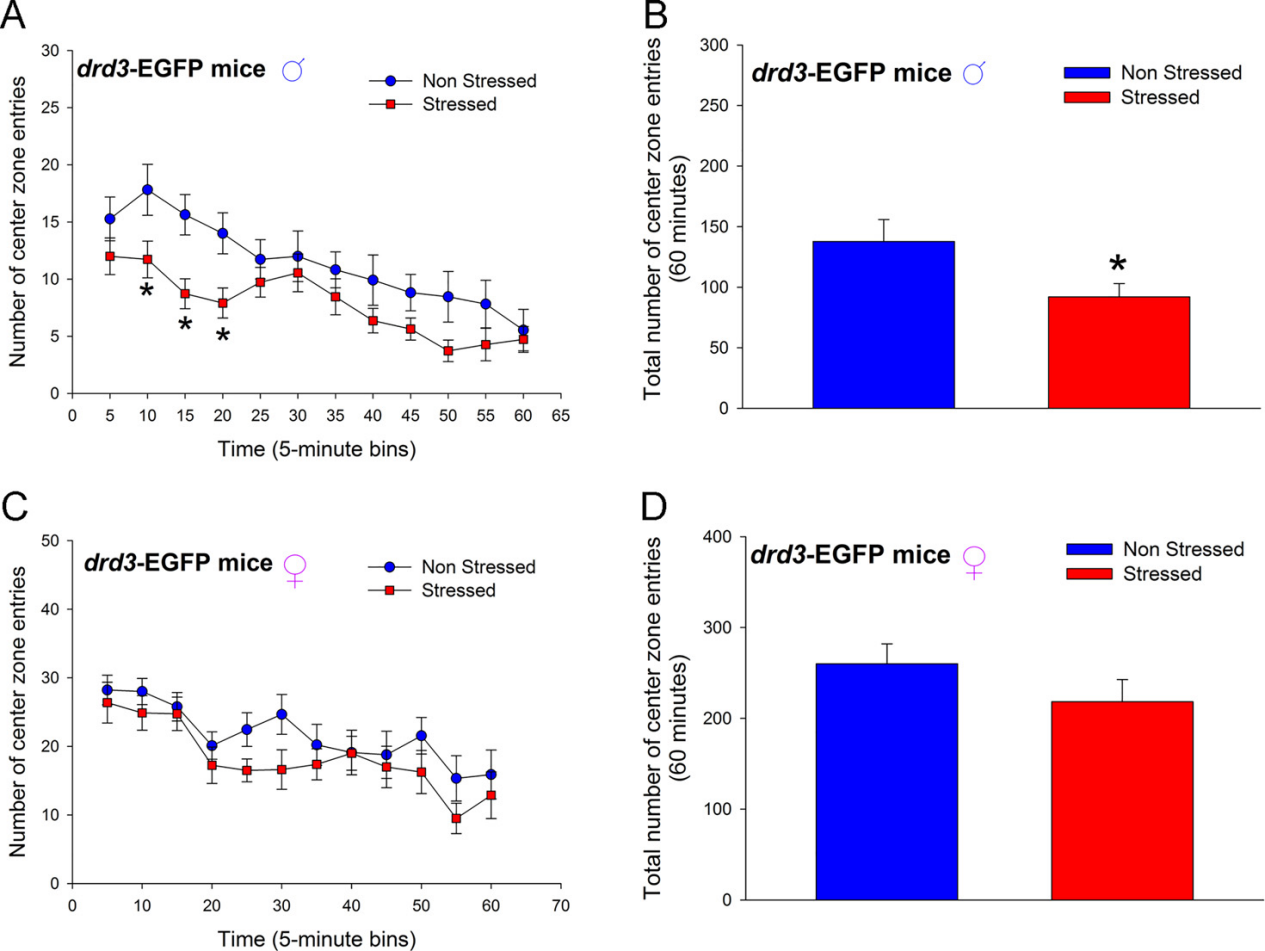
Effect of repeated restraint stress and social isolation during preadolescence on number of center zone entries in an open field arena by adult *drd3*-EGFP mice. Adult male (**A** and **B**) and female (**C** and **D**) *drd3*-EGFP mice were either not stressed (blue, n = 11) or subjected to repeated restraint stress and social isolation during preadolescence (red, n = 11). Number of center zone entries were measured in an Open Field test in adulthood. Significant reduction in number of entries was observed in stressed male *drd3*-EGFP mice during the initial 20 minute period following the placement of the mice in the arena (*, p<0.05, two-way repeated measure ANOVA, post-hoc SNK test; **A**). The total number of center zone entries over the entire 60 minute observation period was also significantly reduced in stressed male *drd3*-EGFP mice (*, p<0.05, Student’s *t*-test). The stress-induced changes in locomotor activity was absent in adult female *drd3*-EGFP mice subjected to preadolescent stress and social isolation (**C** and **D**). Error bars represents ± SEM.

Male *drd3*-EGFP mice that were subjected to preadolescent repetitive restraint stress followed by social isolation exhibited, in adulthood, a significant reduction in number of center zone entries in a novel arena (*p* = 0.043, F_1,220_ = 4.772, two-way repeated measure ANOVA; Fig 2A) wherein, post-hoc SNK test revealed significant difference between the non-stressed and stressed mice only at the 10 min (*q* = 3.647, *p* = 0.013), 15 min (*q* = 4.137, *p* = 0.005) and 20 min (*q* = 3.647, *p* = 0.013) time points. No significant difference in basal center zone entries was observed during the initial 5 min period after the mice were placed in the novel arena (*q* = 1.959, *p* = 0.171). The large decrease in center zone entries during the 10 to 20 min interval resulted in an overall decrease in number of center zone entries over the 60 minute monitoring period (*p* = 0.043, Student’s t-test; Fig 2B). Female *drd3*-EGFP mice that were subjected to preadolescent repetitive restraint stress followed by social isolation did not exhibit, in adulthood, a significant reduction in number of center zone entries in a novel environment (*p* = 0.222, F_1,165_ = 1.635, two-way repeated measure ANOVA; Fig 2C) and, post-hoc SNK test did not reveal significant difference between the non-stressed and stressed mice at any time points. There was also no significant difference in number of center zone entries during the entire 60 minute monitoring period (*p* = 0.222, Student’s t-test; Fig 2D). Comparison of non-stressed male and female *drd3*-EGFP mice showed that the female *drd3*-EGFP mice exhibited a significantly greater number of center zone entries (Fig 2). The transient reduction of locomotor activity in a novel environment and decrease in number of center zone entries in an Open Field test that we observed here in the stressed male *drd3*-EGFP mice is considered an endophenotype of anxiety-related behavior in rodent models according to previous studies [27]. Together the results in Figs 1 and 2, suggest that repetitive restraint stress followed by social isolation during preadolescence results in the development of anxiety-related behaviors in adulthood, specifically in male mice.

### Preadolescent stress-induced adult anxiety-related disorders is mediated by D3 receptors

**Fig 3:**
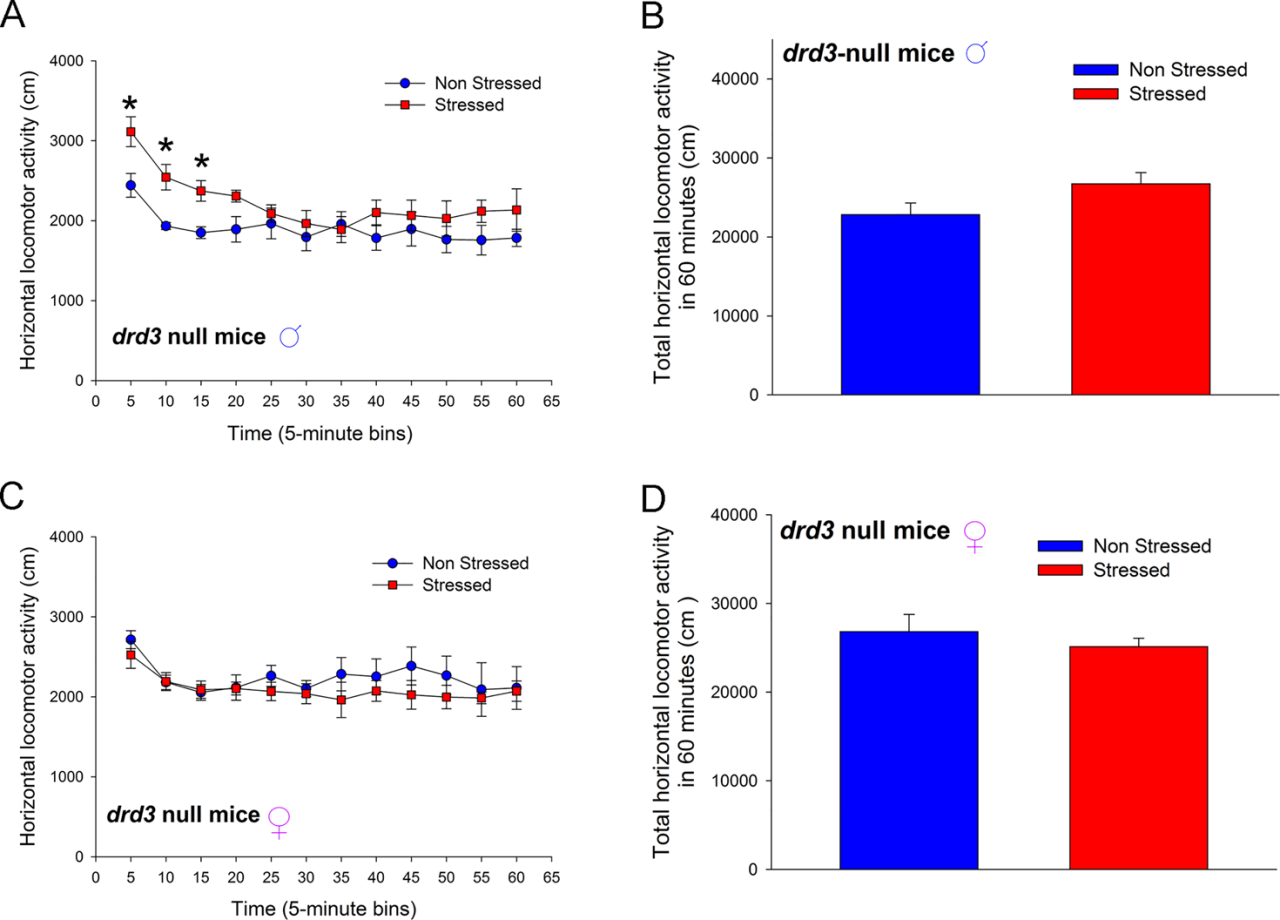
Effect of repeated restraint stress and social isolation during preadolescence on locomotor activity in an open field arena test by adult *drd3*-null mice. Adult male (**A** and **B**) and female (**C** and **D**) *drd3*-null mice were either not stressed (blue, n = 5) or subjected to repeated restraint stress and social isolation during preadolescence (red, n = 5). Horizontal locomotor activity were measured in an Open Field test in adulthood. Compared to non-stressed *drd3*-null male mice, preadolescent stressed male *drd3*-null mice exhibited significant increase in locomotor activity (**A**) only during the initial 15 minute period following the placement of the mice in the arena (*, p<0.05, two-way repeated measure ANOVA, post-hoc SNK test). The total locomotor activity over the entire 60 minute observation period was not significantly altered in stressed male *drd3*-null mice (p>0.05, Student’s *t*-test). The stress-induced changes in locomotor activity was absent in adult female *drd3*-null mice subjected to preadolescent stress and social isolation (**C** and **D**). Error bars represents ± SEM.

To determine the role of dopamine D3 receptor in preadolescent stress-induced adult anxiety-related disorders, we subjected male and female *drd3* null mice to the preadolescent restraint stress and social isolation protocol and assessed anxiety-related behaviors in adulthood. Male *drd3* null mice, lacking the dopamine D3 receptor gene, that were subjected to the preadolescent stress and social isolation (Fig 3A and 3B) did not show a significant reduction in locomotor activity in the Open Field test (*p* = 0.093, F_1,88_ = 3.644, two-way repeated measure ANOVA; Fig 3A). However, post-hoc SNK test revealed significant *increase* in locomotor activity of stressed mice only at the 5 min (*q* = 4.134, *p* = 0.007), 10 min (*q* = 3.762, *p* = 0.014) and 15 min (*q* = 3.225, *p* = 0.032) time points. There was also no significant difference in locomotor activity of the male *drd3* null mice during the entire 60 minute monitoring period (*p* = 0.093, Student’s t-test; Fig 3B). Female *drd3*-null mice that were subjected to preadolescent repetitive restraint stress followed by social isolation did not exhibit, in adulthood, a significant change in locomotor activity in a novel environment (*p* = 0.454, F_1,88_ = 0.618, two-way repeated measure ANOVA; Fig 3C) and, post-hoc SNK test did not reveal significant difference between the non-stressed and stressed mice at any time points. There was also no significant difference in locomotor activity during the entire 60 minute monitoring period (*p* = 0.454, Student’s t-test; Fig 3D).

**Fig 4:**
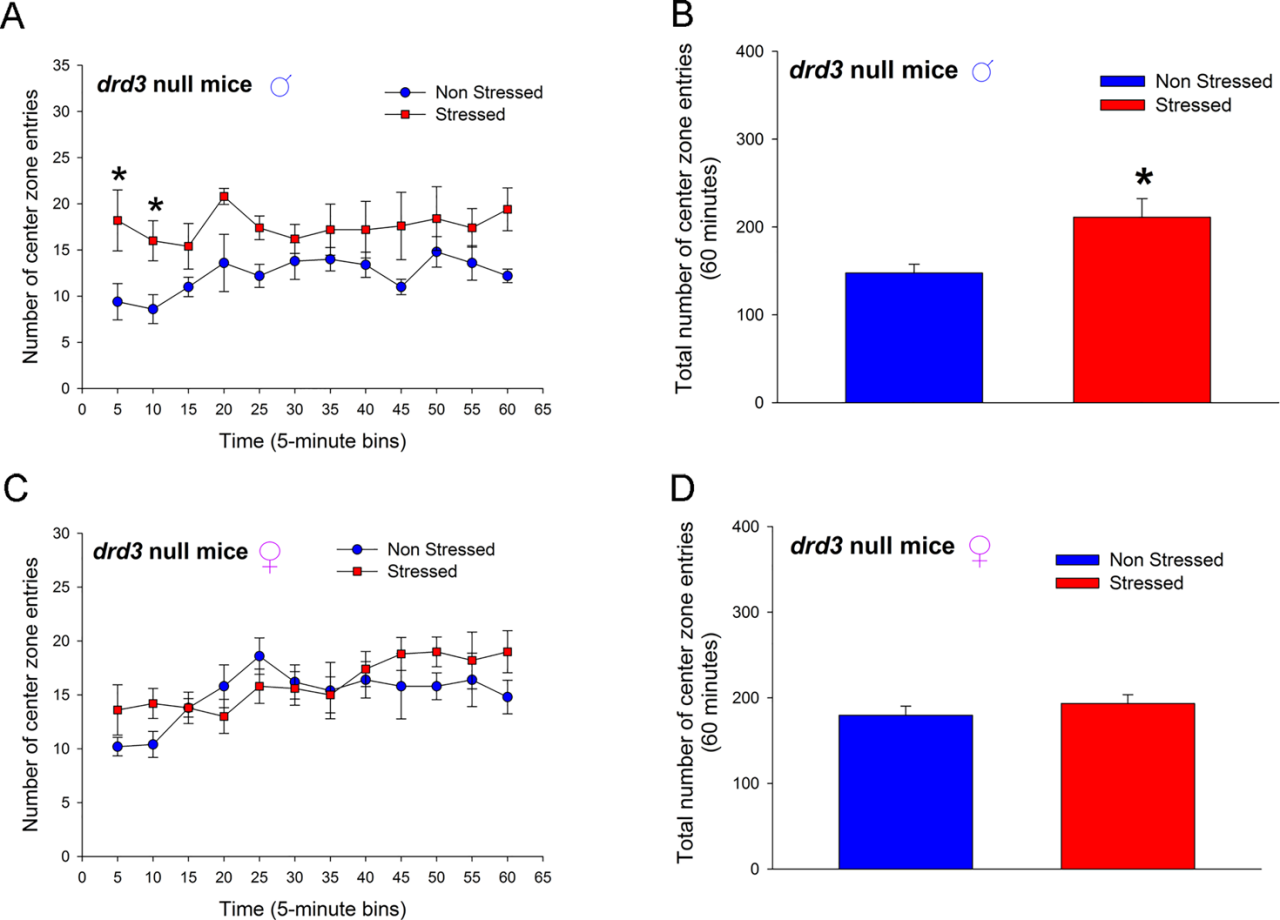
Effect of repeated restraint stress and social isolation during preadolescence on number of center zone entries in an open field arena by adult *drd3*-null mice. Adult male (**A,** and **B**) and female (**C** and **D**) *drd3*-null mice were either not stressed (blue, n = 5) or subjected to repeated restraint stress and social isolation during preadolescence (red, n = 5). Number of center zone entries were measured in an Open Field test in adulthood. Significant increase in number of entries was observed in stressed male *drd3*-null mice during the initial 10 minute period following the placement of the mice in the arena (*, p<0.05, two-way repeated measure ANOVA, post-hoc SNK test; **A**). The total number of center zone entries over the entire 60 minute observation period was also significantly increase in stressed male *drd3*-null mice (*, p<0.05, Student’s *t*-test). The stress-induced changes in locomotor activity was absent in adult female *drd3*-null mice subjected to preadolescent stress and social isolation (**C** and **D**). Error bars represents ± SEM.

Male *drd3*-null mice that were subjected to preadolescent repetitive restraint stress followed by social isolation exhibited, in adulthood, a significant *increase* in number of center zone entries in a novel arena (*p* = 0.026, F_1,88_ = 7.458, two-way repeated measure ANOVA; Fig 4A) wherein, post-hoc SNK test revealed significant difference between the non-stressed and stressed mice only at the 5 min (*q* = 4.077, *p* = 0.006) and 10 min (*q* = 3.428, *p* = 0.02) time points. The large increase in center zone entries during the 5 to 10 min interval resulted in an overall increase in center zone entries over the entire 60 minute monitoring period (*p* = 0.026, Student’s t-test; Fig 4B). Female *drd3*-null mice that were subjected to preadolescent repetitive restraint stress followed by social isolation did not exhibit, in adulthood, a significant change in number of center zone entries in a novel environment (*p* = 0.376, F_1,88_ = 0.877, two-way repeated measure ANOVA; Fig 4C) and, post-hoc SNK test did not reveal significant difference between the non-stressed and stressed mice at any time points. There was also no significant difference in number of center zone entries during the entire 60 minute monitoring period (*p* = 0.376, Student’s t-test; Fig 4D).

**Fig 5:**
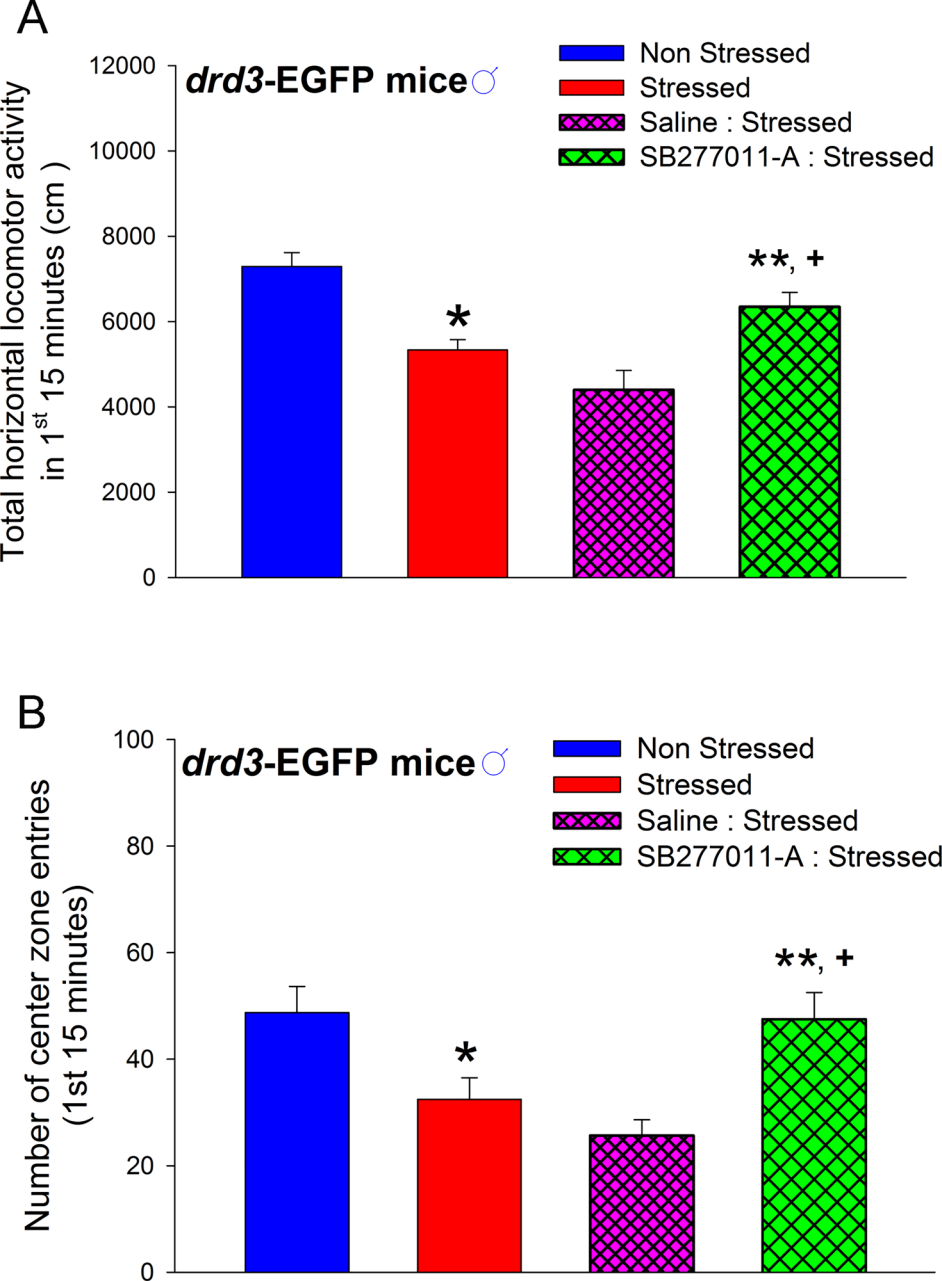
Effect of D3 receptor antagonist on preadolescent stress-induced anxiety-related behaviors in adult *drd3*-EGFP mice. Adult male *drd3*-EGFP mice were either not stressed (blue, n = 11) or subjected to repeated restraint stress and social isolation during preadolescence (red, n = 11). Horizontal locomotor activity (**A**) and number of center zone entries (**B**) were measured in an Open Field test in adulthood. Significant reduction in locomotor activity and center zone entries were observed in stressed *drd3*-EGFP mice (*, p<0.05). Administration of 10 mg/kg SB277011-A (green cross hatch; n = 6) but not saline (pink cross hatch, n = 6) to *drd3*-EGFP mice ten minutes before each restraint stress episode during preadolescence prevented the reduction of locomotor activity (**A**) and center zone entries observed in adulthood (**B**) (**, p<0.001 compared to saline-injected stressed mice and +, p<0.05 compared to non-injected stressed mice). Error bars represents ± SEM.

Consistent with the studies using D3 receptor knock-out mice, the transient reduction in adult locomotor activity and center zone entries in a novel environment was blocked when the preadolescent male mice were systemically administered 10 mg/kg SB277011-A, a D3 receptor-selective antagonist, 10 minutes before each restraint stress episode during the P35-P39 preadolescent period. Note that these antagonist-injected mice were subjected to both restraint stress and subsequent social isolation as in the previous experiments. In addition, administration of the same dose of SB277011-A (10 mg/kg) to naïve *drd3*-EGFP mice did not significantly affect basal locomotor activity (S1 Fig). The transient reduction in locomotor activity during the initial 15 minutes in the novel arena was significantly attenuated in adult male mice that were administered SB277011-A before each restraint stress episode during preadolescence (*p*<0.001, F_3,30_ = 14.1, one-way ANOVA, SNK post-hoc test; Fig 5A). Similarly, the transient reduction in center zone entries was significantly blocked in male mice that were administered SB277011-A prior to each restraint stress episode during preadolescence (*p* = 0.004, F_3,30_ = 5.15, one-way ANOVA, SNK post-hoc test; Fig 5B).

Together the results in Figs 3, 4 and 5 strongly suggest that the dopamine D3 receptor is essential for causing the adult anxiety-related behaviors in male mice that were subjected to preadolescence repeated restraint stress and social isolation.

### Preadolescence restraint stress and social isolation causes depression-related disorders in adult male and female mice

**Fig 6:**
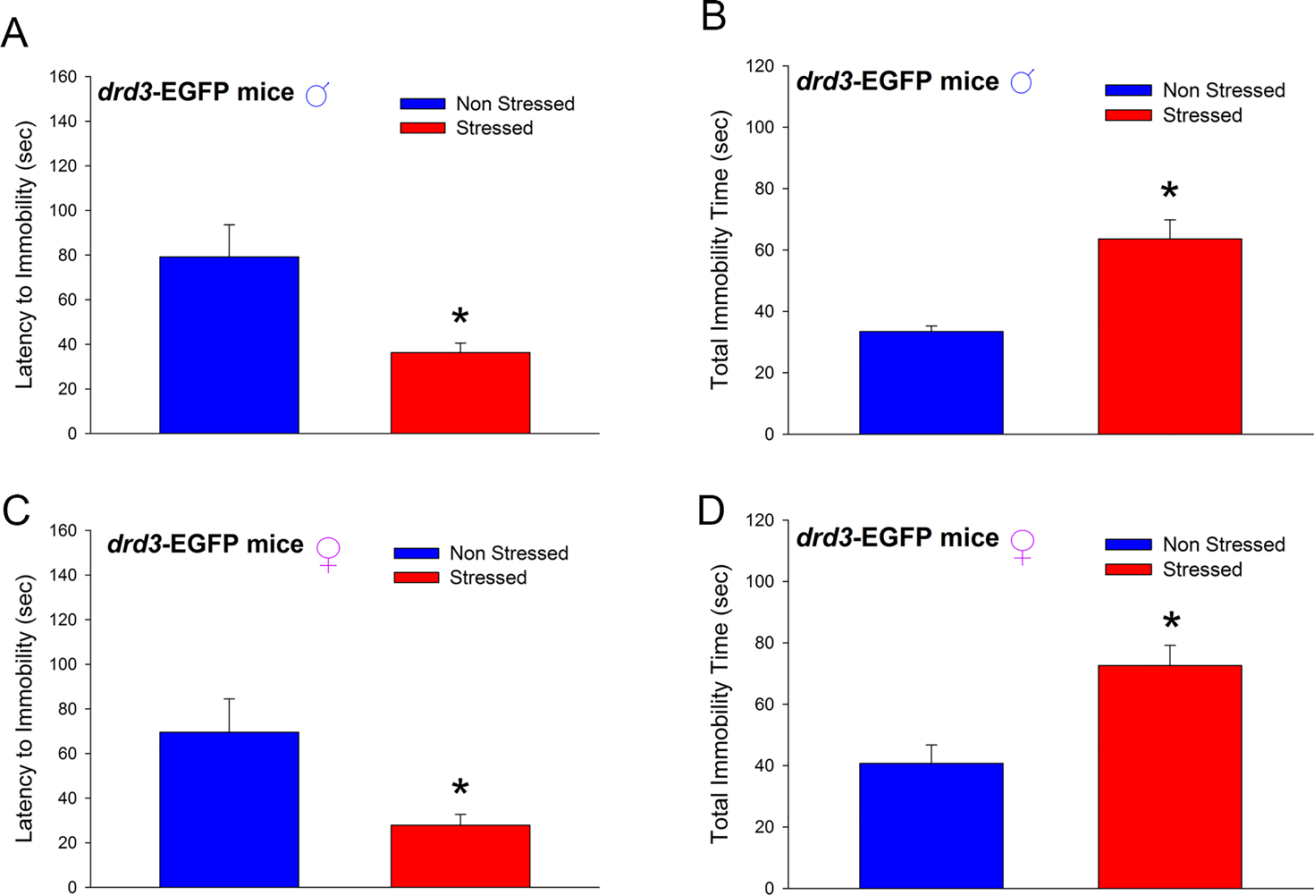
Effect of repeated restraint stress and social isolation during preadolescence on depression-related behaviors in adult *drd3*-EGFP mice. Adult male (**A** and **B**) and female (**C** and **D**) *drd3*-EGFP mice were either not stressed (blue, n = 7) or subjected to repeated restraint stress during preadolescence (red, n = 7). Latency to immobility (**A** and **C**) and total immobility time (**B** and **D**) were measured in the Porsolt Forced Swim test. Significant reduction in latency to immobility and increase in total immobility time were observed in adult male and female *drd3*-EGFP mice that were subjected to repeated restraint stress during preadolescence (*, p<0.001). Error bars represents ± SEM.

In adulthood, male *drd3*-EGFP mice that were subjected to preadolescence repetitive restraint stress and social isolation exhibited decreased latency to immobility (*p*<0.001, U = 8.5, Mann-Whitney Rank Sum test; Fig 6A) and increased total immobility time (*p*<0.001, U = 12.5, Mann-Whitney Rank Sum test; Fig 6B), in the Porsolt Forced Swim test, compared to non-stressed littermates. In the Porsolt Forced Swim test, reduction in latency to immobility and increased total immobility time, result from behavioral despair which is an endophenotype of depression-related behaviors in animal models. Interestingly, in contrast to the male-specific effect on anxiety shown in Figs 1 and 2, the preadolescent stress-induced changes in latency to immobility and total immobility (*p* = 0.004, U = 3.0, Mann-Whitney Rank Sum test and *p* = 0.003, Student’s t-test, respectively) were also observed in adult female *drd3*-EGFP mice subjected to the preadolescent restraint stress and social isolation treatment (Fig 6C and 6D). Together these results suggest that repetitive restraint stress during preadolescence and social isolation results in the development of depression-related behaviors in adulthood, in both male and female mice.

### Preadolescent stress-induced adult depression-related disorders is mediated by D3 receptors

**Fig 7:**
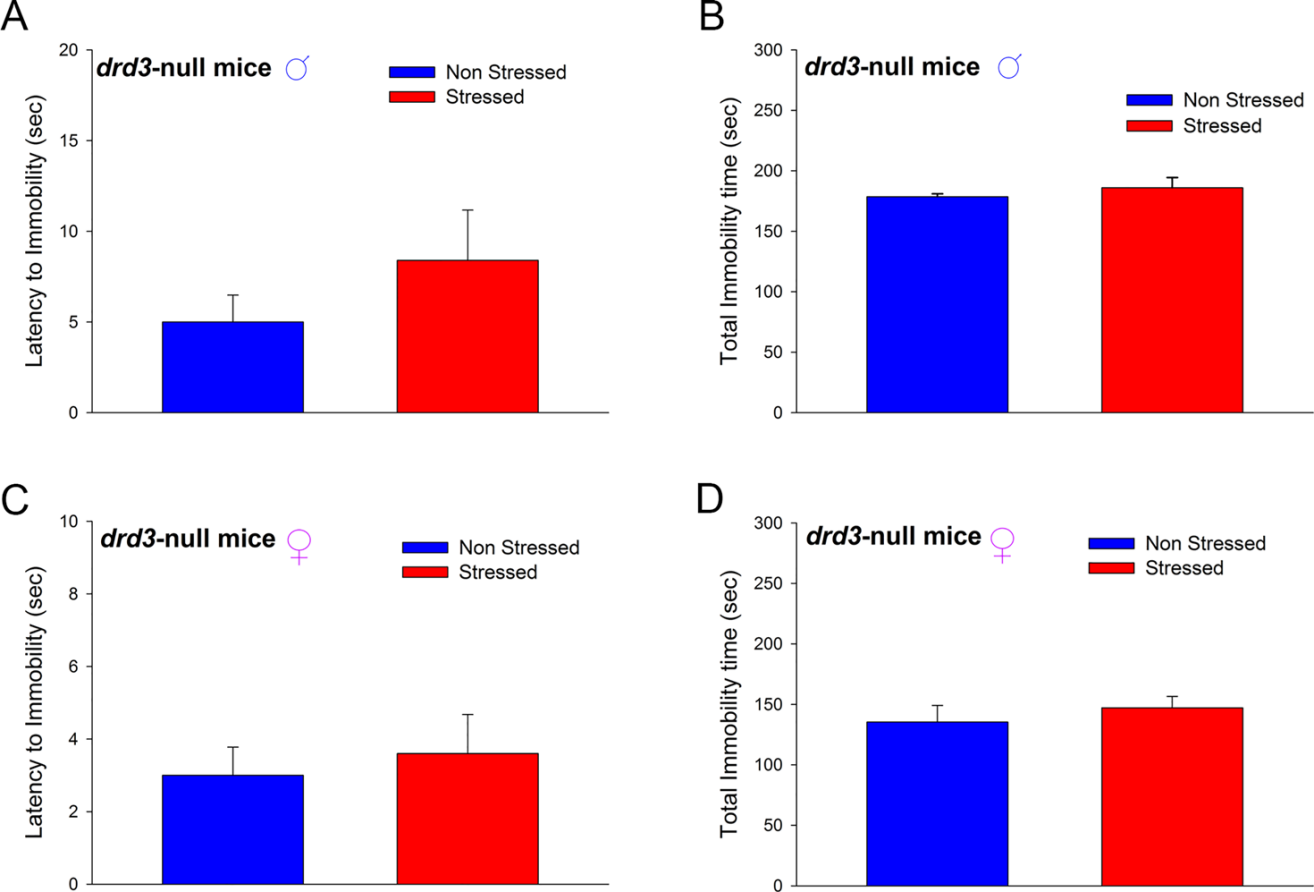
Effect of D3 receptor genetic ablation on preadolescent stress-induced depression-related behaviors in adult mice. Male (**A** and **B**) and female (**C** and **D**) *drd3*-null mice, in adulthood, did not show significant difference in latency to immobility (**A** and **C**) and total immobility time (**B** and **D**) regardless of whether they were subjected preadolescent repeated restraint stress and social isolation. N = 6 animals. Error bars represents ± SEM.

To determine the role of dopamine D3 receptor in preadolescent stress-induced adult depression-related disorders, we subjected male and female *drd3* null mice to the preadolescent restraint stress and social isolation protocol and assessed depression-related behaviors in adulthood. In adulthood, male *drd3*-null mice that were subjected to preadolescence repetitive restraint stress and social isolation exhibited no significant difference in latency to immobility (*p* = 0.31, Student’s t-test; Fig 7A) and total immobility time (*p* = 0.69, U = 10, Mann-Whitney Rank Sum test; Fig 7B), in the Porsolt Forced Swim test, compared to non-stressed littermates. Similarly, adult female *drd3*-null mice subjected to the preadolescent restraint stress and social isolation treatment exhibited no significant difference in latency to immobility (*p* = 0.663, Student’s t-test; Fig 7C) and total immobility time (*p* = 0.421, U = 8.5, Mann-Whitney Rank Sum test; Fig 7D), in the Porsolt Forced Swim test, compared to non-stressed littermates.

**Fig 8:**
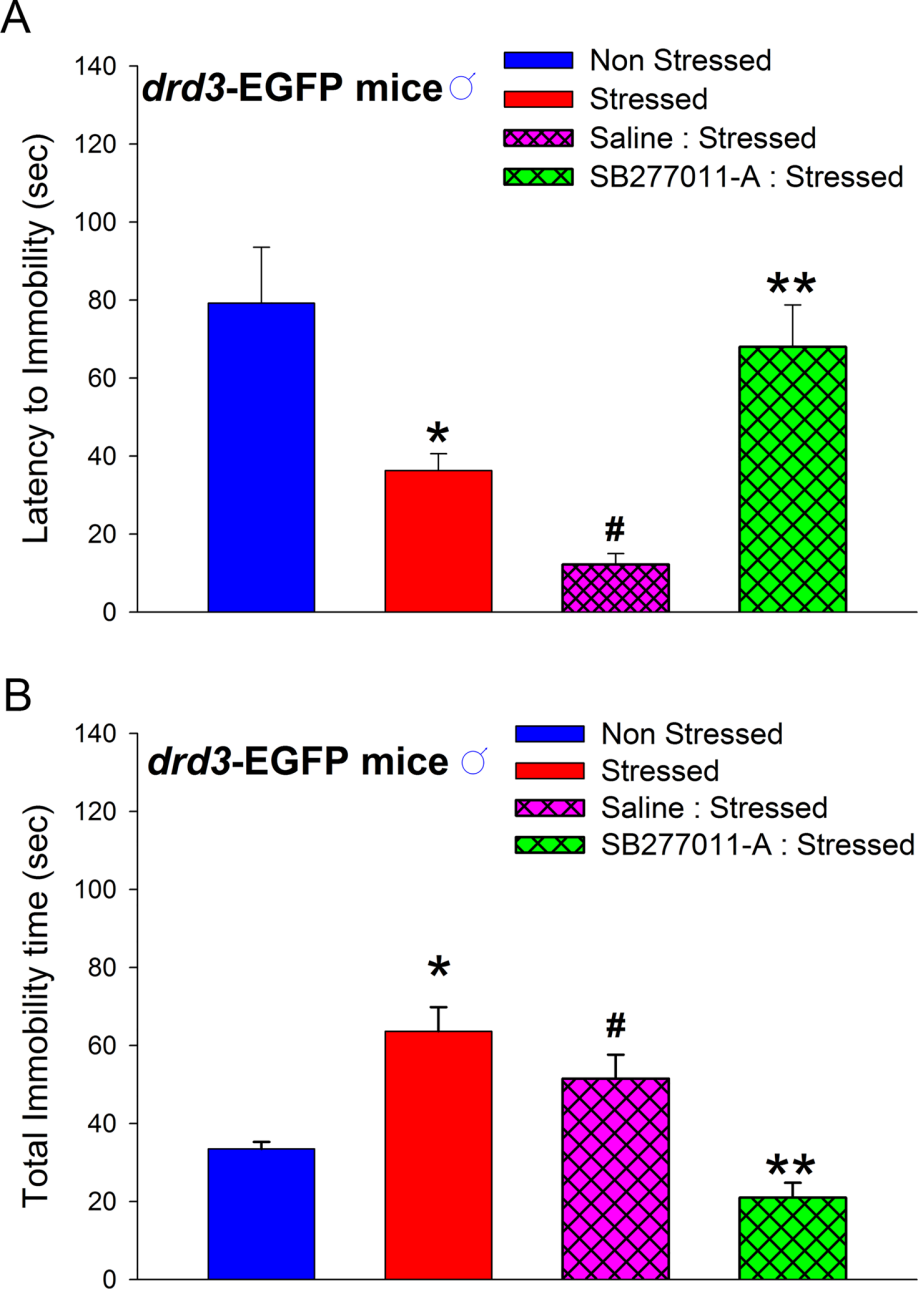
Effect of D3 receptor antagonism on preadolescent stress-induced depression-related behaviors in adult mice. Administration of 10 mg/kg SB277011-A (n = 6) but not saline (n = 6) to male *drd3*-EGFP mice ten minutes before each restraint stress episode during preadolescence prevented the reduction in latency to immobility (**A**) and increase in total immobility time (**B**) in adulthood (**, p<0.001 compared to saline-injected stressed mice and non-injected stressed mice). Significant differences were also observed between non-stressed mice and saline-injected stressed mice (#, p<0.05). Error bars represents ± SEM.

The depression-related behaviors observed in the adult mice that were subjected to repetitive restraint stress during preadolescence and social isolation were prevented if the preadolescent *drd3*-EGFP mice were systemically administered 10 mg/kg SB277011-A, a D3 receptor-selective antagonist, 10 minutes before each restraint stress episode during the P35-P39 preadolescent period (Fig 8A and 8B). Note that these antagonist-injected mice were subjected to both restraint stress and subsequent social isolation as in the previous experiments. In addition, administration of the same dose of SB277011-A (10 mg/kg) to naïve *drd3*-EGFP mice did not significantly affect basal locomotor activity (S1 Fig). The decrease in latency to immobility, in adulthood, was significantly attenuated in mice that were administered SB277011-A before each preadolescent restraint stress episode (*p* = 0.01, q = 4.4, one-way ANOVA, post-hoc SNK test; Fig 8A). Similarly, the increase in total immobility time, in adulthood, was significantly reduced in mice that were administered SB277011-A prior to each restraint stress episode during preadolescence (*p* = 0.006, q = 4.76, one-way ANOVA, post-hoc SNK test; Fig 8B). These results suggest that the dopamine D3 receptor plays a central role in the etiology of adult depression-related disorders that are caused by preadolescent stress and social isolation.

### Adult mice subjected to preadolescent stress and social isolation exhibit sex-specific differences in expression and function of D3 receptors in the limbic regions

**Fig 9:**
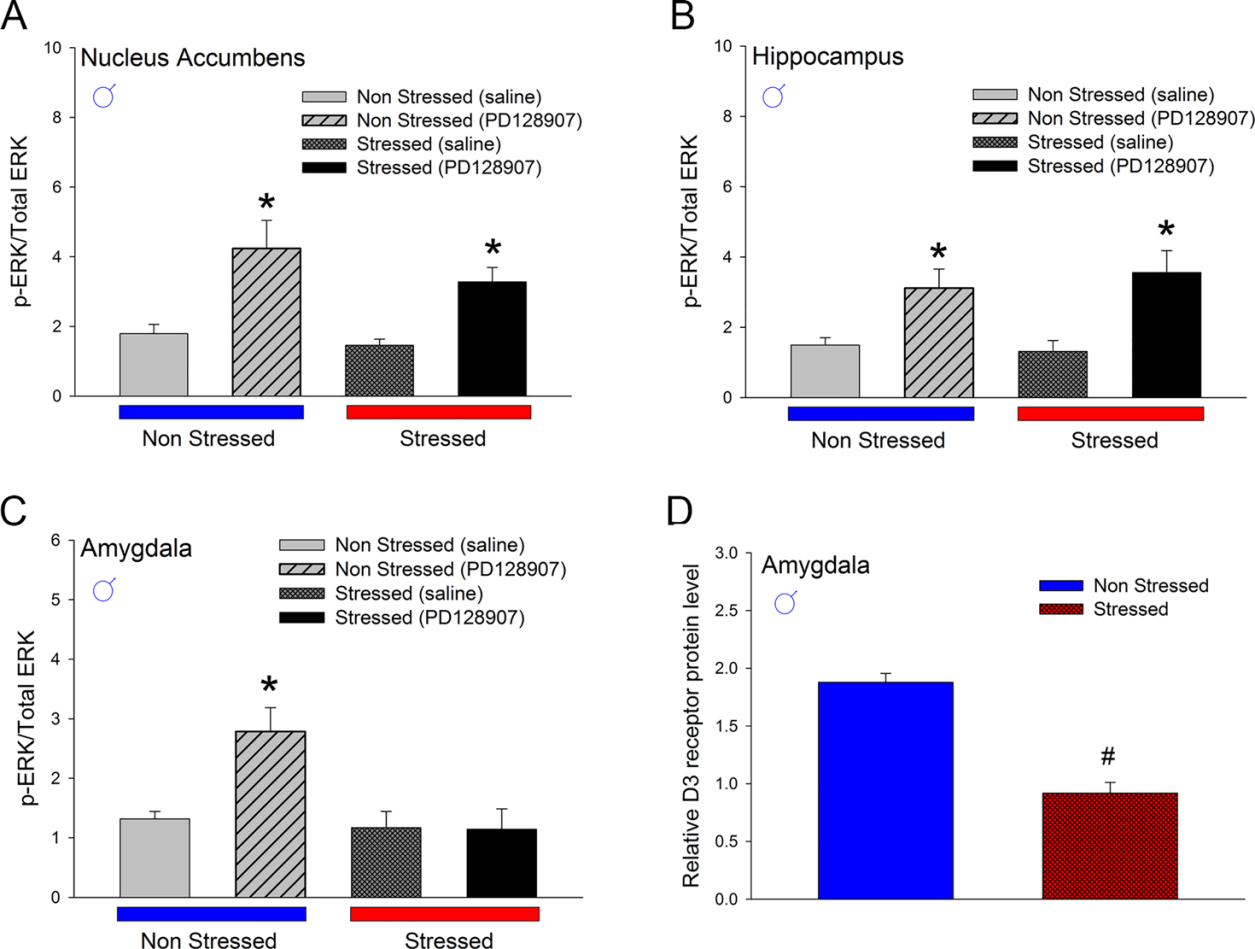
Effect of repeated restraint stress and social isolation during preadolescence on function and expression of dopamine D3 receptors in three limbic brain regions of adult male *drd3*-EGFP mice. Male *drd3*-EGFP mice (**A-D,** n = 5 per treatment group) were either not stressed (blue) or subjected to preadolescent restraint stress and social isolation (red). In adulthood, the two groups of mice were administered saline or 0.05 mg/kg PD128907, s.c., a D3 receptor agonist. The three brain regions nucleus accumbens (**A**), hippocampus (**B**) and amygdala (**C** and **D**) were harvested ten minutes after the injection. The levels of phosphorylated ERK and total ERK were measured using western blot and expressed as a ratio (**A-C**). A significant increase in PD128907-induced ERK phosphorylation was observed in the nucleus accumbens and hippocampus of non-stressed and stressed mice but only in the amygdala of non-stressed mice (*, p<0.05). There was also a significant reduction of D3 receptor protein levels in the amygdala of stressed mice (**D**, # p<0.05). Error bars represents ± SEM.

**Fig 10:**
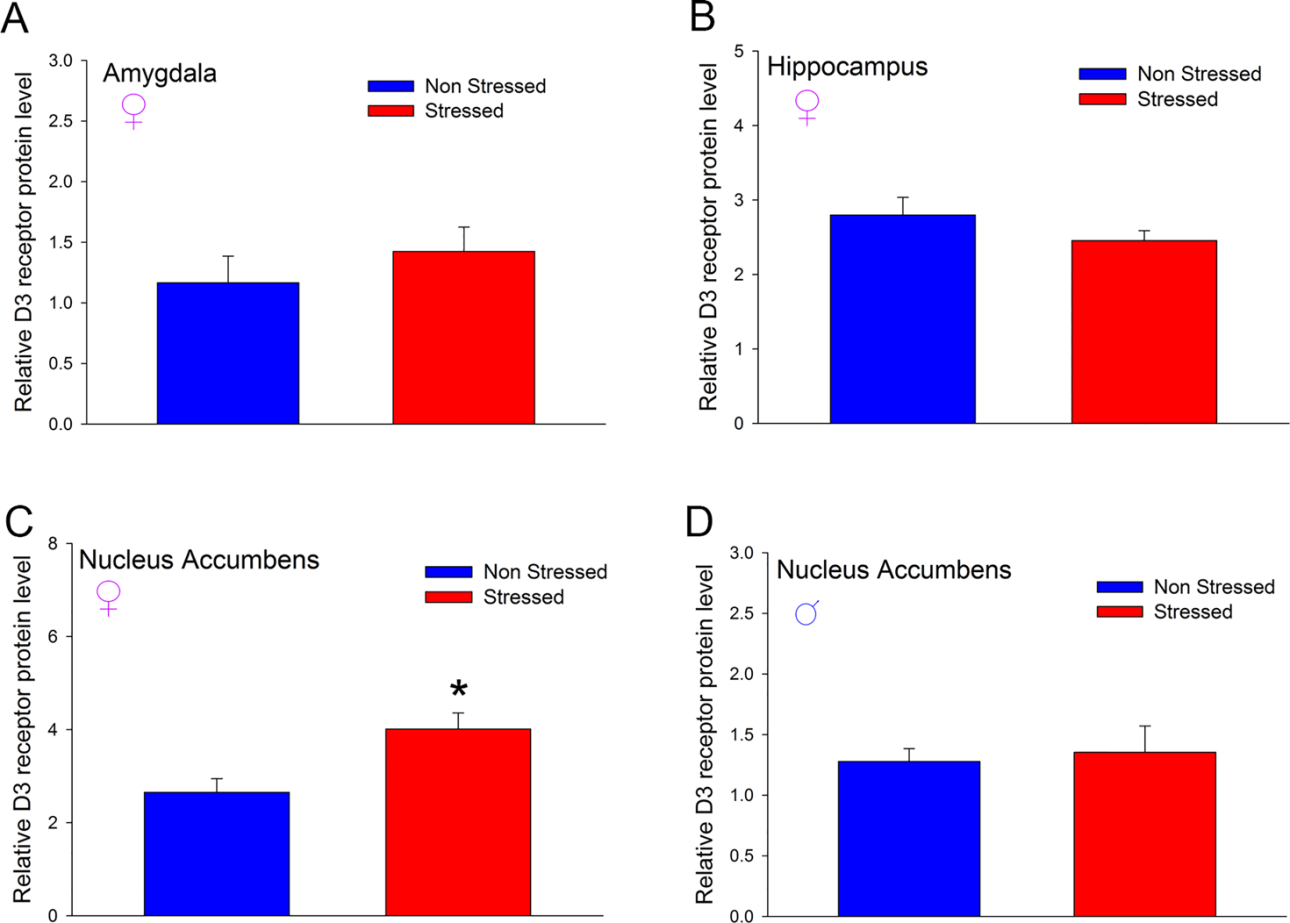
Effect of repeated restraint stress and social isolation during preadolescence on expression of dopamine D3 receptors in three limbic brain regions of adult *drd3*-EGFP mice. Female *drd3*-EGFP mice (**A-C,** n = 5 per treatment group) and male *drd3*-EGFP mice (D, n = 5 per treatment group) were either not stressed (blue) or subjected to preadolescent restraint stress and social isolation (red). In adulthood, the three brain regions, amygdala (**A**), hippocampus (**B**) and nucleus accumbens (**C** and **D**) were harvested. The levels of D3 receptor protein were measured using western blot and normalized to total proteins levels in each lane (detected by amido black staining of the blot). A significant increase in D3 receptor expression was observed in the nucleus accumbens of female stressed mice (*, p<0.05, Student’s *t*-test). Error bars represents ± SEM

Next, we compared the signaling function of dopamine D3 receptor in adult male non-stressed and stressed *drd3*-EGFP mice in nucleus accumbens, hippocampus and amygdala. We have previously shown that 0.05 mg/kg PD128907 selectively activates the D3 receptor-mitogen activated protein kinase signaling pathway in *drd3*-EGFP mice resulting in increased phosphorylated ERK protein levels [13]. The results in Fig 9 show that while activation of MAPK pathway by D3 receptor in the nucleus accumbens (Fig 9A; *p*<0.001, F_1,16_ = 19.646, two-way ANOVA, post-hoc SNK test) and hippocampus (Fig 9B;; *p* = 0.001, F_1,14_ = 17.03, two-way ANOVA, post-hoc SNK test) is unaffected by preadolescent stress, there is a loss of D3 receptor-MAPK signaling function in the amygdala of adult male *drd3*-EGFP mice that were subjected to preadolescent repetitive restraint stress and social isolation (Fig 9C; *p* = 0.953, q = 0.09, two-way ANOVA, post-hoc SNK test). This loss of D3 receptor signaling function in the amygdala correlated with a significant decrease in D3 receptor protein expression in the amygdala of adult male *drd3*-EGFP mice subjected to preadolescent stress and social isolation (Fig 9D; *p*<0.001, Student’s t-test). Given our observation that preadolescence stress and social isolation caused anxiety- and depression-related disorders in adult male mice but only depression-related disorders in adult female mice, we measured the expression of dopamine D3 receptor in control and stressed adult female *drd3-*EGFP mice in amygdala, hippocampus and nucleus accumbens (Fig 10). Interestingly, the result showed no difference in D3 receptor expression in the amygdala or hippocampus (Fig 10A and 10B); however, there was a significant *increase* in D3 receptor expression in the nucleus accumbens of female mice (*p*<0.01, Student’s *t*-test; Fig 10C and S2 Fig) and no change in D3 receptor expression in nucleus accumbens of male mice (Fig 10D). Together these results suggest that the decrease of D3 receptor expression and loss of D3 receptor signaling function in the amygdala might contribute to the adult anxiety- and depression-related behaviors in male mice that were subjected to preadolescent stress and social isolation. In contrast, an increase in expression of D3 receptor in the nucleus accumbens of female mice subjected to preadolescent stress and social isolation might contribute to depression-related disorders in adulthood.

## Discussion

The results in this paper show that repeated restraint stress episodes over a five-day period during preadolescence followed by social isolation causes sexually dimorphic anxiety and depression-related disorders in adult mice. In this study, we used two measures of anxiety-related behavior (1) reduction in locomotor activity during the initial period in a novel environment and (2) reduction in number of center zone entries or thigmotaxis, both of which have been previously used to assess anxiety–related disorders in rodents [27]. Given the overall reduction in locomotor activity of male mice in the Open Field test, there is a possibility that preadolescent stress affects motor function; therefore, in future studies, the anxiety-related behaviors observed in male *drd3*-EGFP mice will have to be validated by other methods for assessing anxiety-related disorders such as elevated plus maze. The current study also used an experimental paradigm that included repetitive restraint stress followed by social isolation. Future studies will distinguish the effect of restraint stress from social isolation stress.

Studies have shown that the activity of dopaminergic system in the CNS including the levels of dopamine and dopamine receptors peaks during preadolescence [28]. Our results raise the possibility that the repeated restraint stress alters the normal dopaminergic developmental program in stress-sensitive regions such as the amygdala and nucleus accumbens [11]. Among the limbic regions implicated in depression- and anxiety-related disorders, we observed a loss of D3 receptor expression and signaling function in the amygdala of male mice and an increase in expression of D3 receptors in the nucleus accumbens of female mice. Alterations in environmental and social stimulation during preadolescence have been shown to increase dopaminergic projection in the basolateral amygdala, a region involved in affective behaviors [11, 12, 21, 29–32]. We have previously used the *drd3*-EGFP reporter mice and shown that during normal postnatal brain development, the D3 receptor undergoes a switch in co-expression pattern from GABAergic cells in preadolescence to glutamatergic cells in adults, specifically in the amygdala [22]. It remains to be determined if repeated restraint stress during preadolescence followed by social isolation, disrupts the developmental switch in D3 receptor co-expression in the amygdala, giving rise to the loss of D3 receptor expression and function in adult mice. Recent studies in rats have shown that the dopamine D3 receptor in the basolateral amygdala regulates GABAergic neurotransmission and modulates anxiety-like behavior [21]. In a recent single cell analysis study, we reported that majority of the cells expressing D3 receptors in the adult mouse nucleus accumbens and amygdala are GABAergic and glutamatergic, respectively [22]. Given the decrease in D3 receptor expression in the amygdala of adult males subjected to preadolescence stress and increase in its expression in the nucleus accumbens of adult females subjected to preadolescence stress, we speculate that, as an inhibitory dopamine receptor, the mice that experienced preadolescent stress and social isolation might have a reduction of inhibitory control in the excitatory glutamatergic cells in the amygdala and an increase of inhibitory control in inhibitory GABAergic cells in the nucleus accumbens. The net result would be increased excitability of amygdala in male mice and increased excitability of nucleus accumbens in female mice which, might contribute to anxiety- and depression-related behaviors, respectively, in the two sexes. In addition, in the same single cell analysis study, we reported that the co-expression pattern of D3 receptors with D1 and D2 dopamine receptors in the amygdala and nucleus accumbens is very different between adult male and females [22]. The difference in co-expression pattern might also contribute to the sexual dimorphism in anxiety- and depression-related behaviors in the stressed mice.

Among dopamine receptor subtypes, the D3 receptor has the highest affinity for dopamine and many clinically used ligands and also exhibits unique signaling properties [13]. Our results raise the possibility that repeated activation of D3 receptor by clinically administered ligands, which increase dopamine levels or act as direct D3 receptor ligands, during preadolescence might lead to development of anxiety- and depression-related disorders in adulthood. Indeed, administration of dopaminergic drugs such as methylphenidate (e.g. Ritalin®) to children and rodents have been reported to result in the development of psychiatric disorders in adulthood [33, 34]. Adult D3 receptor null mice have also been shown to be resistant to the behavioral dysfunction that results from immobilization stress [18–20]. The results of our current studies with D3 receptor null mice further underscore the critical role of D3 receptor in the etiology of the psychiatric disorders. Interestingly, male but not female, D3 receptor null mice were more susceptible to preadolescent stress and social isolation, as seen by their transient hyperexcitability when placed in a novel environment (Figs 2 and 3). These observations suggest that systemic reduction in D3 receptor expression and function, either due to genetic causes or pharmacological treatment, when coupled with repetitive emotional stress and social isolation during adolescence might contribute to male psychiatric disorders with hyperexcitable phenotypes.

Interestingly, in this preadolescent restraint stress model, the adult male mice exhibit both anxiety and depression-related behaviors whereas female mice only exhibit depression-related behaviors. Coincidently, adult male mice stressed during preadolescence exhibited a reduction in D3 receptor expression and function in the amygdala but adult female mice stressed during preadolescence showed an increased expression of the D3 receptor in the nucleus accumbens. It remains to be seen if these changes in D3 receptor expression directly contribute to the behavioral dysfunction. We also observed that non-stressed female *drd3*-EGFP mice exhibited greater number of center zone entries compared to non-stressed male *drd3*-EGFP mice, suggesting that female mice exhibit less anxiety-like behaviors in a novel environment. Sex-specific differences in induction of anxiety and depression-related behaviors have been previously reported and likely involves the differential effects of hormones during sexual maturation [35]. Regardless, the manifestation of these psychiatric disorders in both sexes required functional dopamine D3 receptors as both pharmacological antagonism and genetic ablation of D3 receptor prevented the preadolescent stress-induced adult disorders. Taken together, our results suggest that repetitive restraint stress during preadolescence reduces the expression and function of D3 receptors in the amygdala of adult males and increases the expression of D3 receptors in the nucleus accumbens of adult female mice which, might contributes to the etiology of adult psychiatric disorders. This differential sex- and brain region-specific changes in D3 receptor expression might be therapeutically relevant when developing dopamine receptor ligands for treating the two different types of psychiatric disorders in males and females.

## Conclusions

There is increasing recognition that chronic or repeated stress during childhood can alter post-natal neurodevelopment and lead to psychiatric disorders in adulthood. In this study, we recapitulated the clinical observations using a mouse model that exhibits preadolescent stress-induced adult psychiatric disorders. Our results suggest that the dopamine D3 receptor is centrally involved in the etiology of preadolescent stress induced psychiatric disorders, making the receptor a potential therapeutic target.

## Supporting Information

S1 Fig Effect of the dose of D3 receptor antagonist used in this study on basal locomotor activity in *drd3*-EGFP mice. In an open field test conducted for 60 minutes, *drd3*-EGFP mice administered 10 mg/kg SB277011-A (red; n = 6) to did not show significant difference in total basal locomotor activity when compared to saline-injected mice (black; n = 6). Error bars represents ± SEM.
(TIF)

S2 Fig Representative western blot showing the expression of D3 receptor protein in the nucleus accumbens from six adult female mice, three of whom were not stressed (NS) and three were subjected preadolescent stress and social isolation (St). The D3 receptor protein was detected using a D3 receptor rabbit monoclonal antibody (Abcam® catalog # ab155098)
(TIF)
