# Supplementary figures and images for: Dopamine D3 Receptor Mediates Preadolescent Stress-Induced Adult Psychiatric Disorders
Source: PLoS One. 2015 Nov 30;10(11):e0143908. doi: 10.1371/journal.pone.0143908 (PMC4664486; doi:10.1371/journal.pone.0143908)

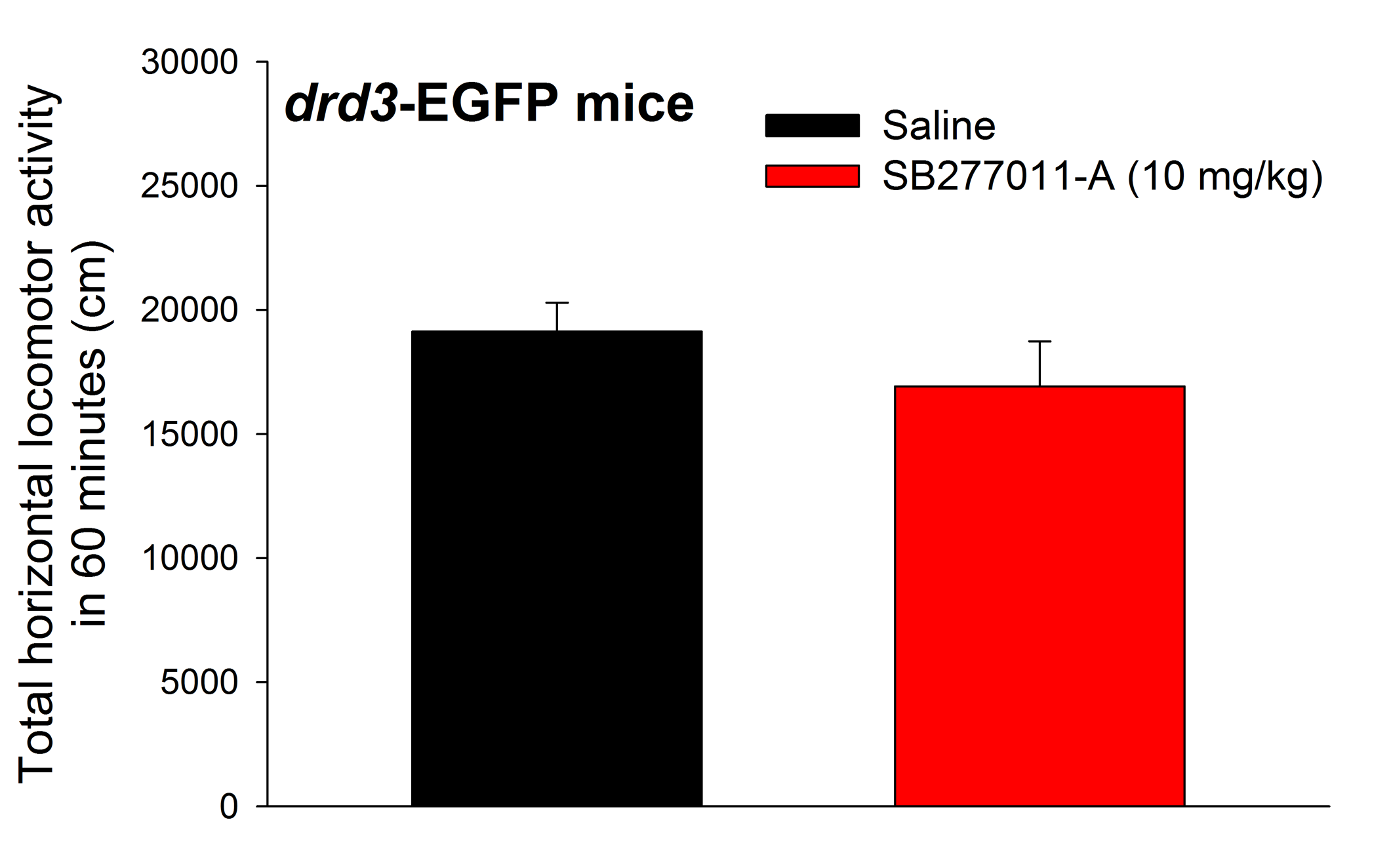

Supplement: S1 Fig — In an open field test conducted for 60 minutes, drd3-EGFP mice administered 10 mg/kg SB277011-A (red; n = 6) to did not show significant difference in total basal locomotor activity when compared to saline-injected mice (black; n = 6). Error bars represents ± SEM. (TIF) [file pone.0143908.s001.tif]

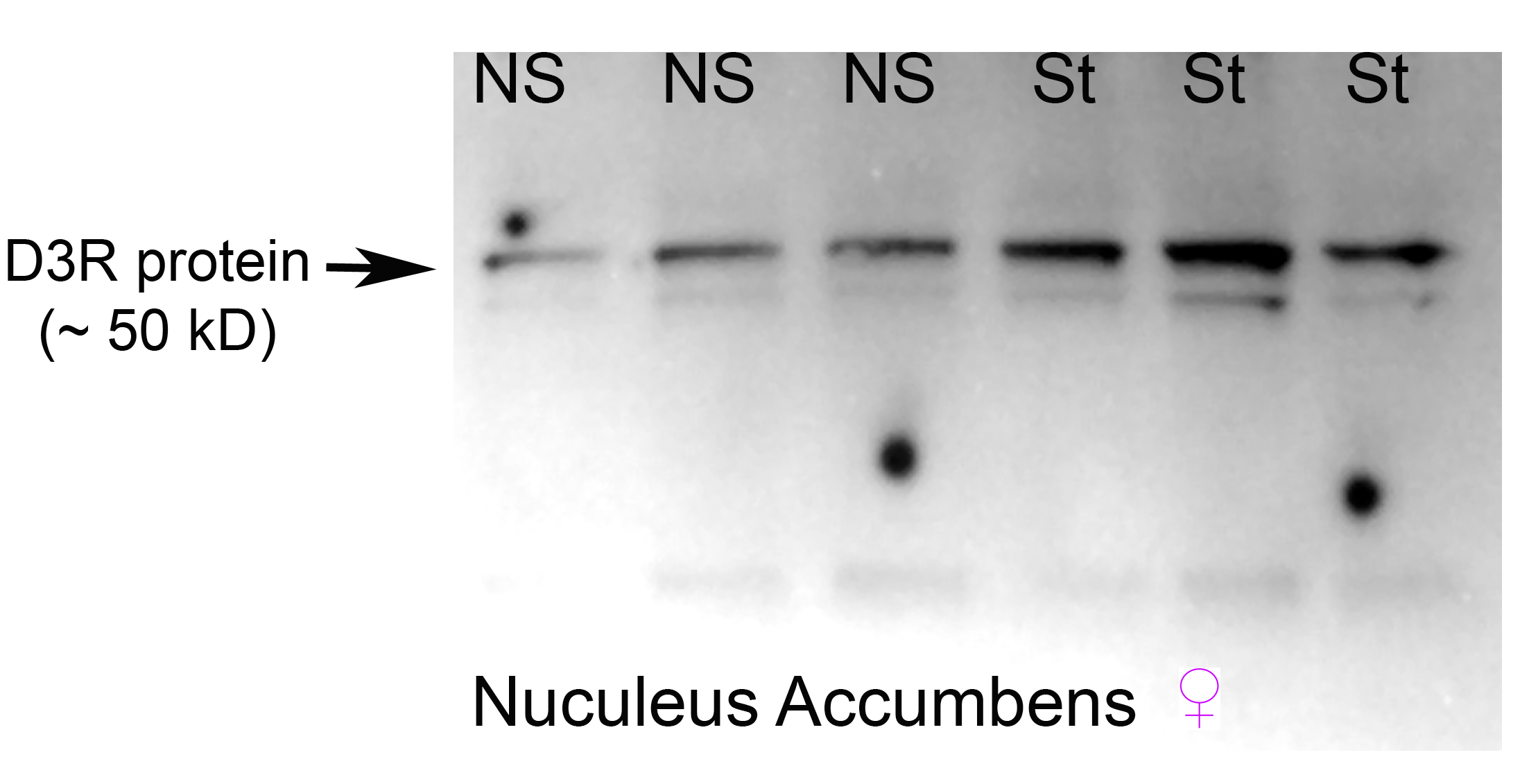

Supplement: S2 Fig — The D3 receptor protein was detected using a D3 receptor rabbit monoclonal antibody (Abcam® catalog # ab155098) (TIF) [file pone.0143908.s002.tif]
